# The Natural Emergence of (Bio)Semiosic Phenomena

**DOI:** 10.1007/s12304-015-9241-4

**Published:** 2015-05-27

**Authors:** J. H. van Hateren

**Affiliations:** Johann Bernouilli Institute for Mathematics and Computer Science, University of Groningen, P.O. Box 407, 9700 AK Groningen, The Netherlands

**Keywords:** Agency, Meaning, Emergence, Semiotics, Peirce, Causation

## Abstract

Biological organisms appear to have agency, goals, and meaningful behaviour. One possibility is that this is mere appearance, where such properties are not real, but only ‘as if’ consequences of the physiological structure of organisms. Another possibility is that these properties are real, as emerging from the organism's structure and from how the organism interacts with its environment. Here I will discuss a recent theory showing that the latter position is most likely correct, and argue that the theory is largely consistent with the basics of the field of biosemiotics. The theory can be represented as a triad that resembles the semiotic triad proposed by Peirce, which connects a sign with its object through a process of interpretation. In the theory presented, the sign is an internalized version of fitness (i.e., expected reproductive rate) which refers to the true fitness through a feedback loop that in effect produces interpretation. The feedback loop entangles deterministic and stochastic forms of causation in such a way that genuine agency, goal-directedness, and their associated meaning emerge. It produces a strong form of emergence not reducible to its constituents. The result is that novel phenomena arise that are real and necessary components for a complete understanding of living organisms.

## Introduction

A biological organism may be seen as a purely material system driven by environmental factors and by the organism's genetic and physiological structure. But it may also be seen as an individual with agency and goals. A basic question that has been haunting biological thinking for a long time is whether the second view is a mere consequence of the first view, or whether it adds something extra. The ‘mere consequence’ idea implies that it is enough to study an organism's structure and physiology in as much detail as possible. Such a detailed analysis will then eventually show that agency and goals are not real but only apparent, in an ‘as if’ kind of way. On the other hand, the ‘adds something extra’ idea seems to require ingredients that have no counterpart in the non-living material world. Introducing such ingredients on an ad hoc basis is an unattractive proposition.

A way out for the ‘adds something extra’ view may be the concept of emergence, the idea that new properties may arise from specific configurations of matter. For example, certain spherical objects with sufficient hardness obtain the property that they can roll on a plain, and the property of rollability may then be seen as emergent. However, that would be a property that is fully predictable once the properties of the material and the configuration are specified, and rollability is not radically different from other mechanical properties that are known to exist. The problem with agency and goals is that they do seem to be radically different from anything else in nature. If agency and goals are really emergent, it needs to be shown in which specific way they can emerge and why it is plausible that they arise in the radically new form they do.

In this article, I discuss a theory (van Hateren 2015) that can indeed let agency and goals emerge from components that lack those properties. I specifically put this theory within the context of the field of biosemiotics, which addresses similar issues, and show that it matches quite well with the main ideas of that field. Moreover, I argue that the emerging properties are fundamentally new and cannot be reduced to (or replaced by) a description of components and their configuration.

The article is organized as follows. The theory presented depends on Darwinian mechanisms, including modern additions as in an extended synthesis (see e.g., Pigliucci and Müller 2010), but also the crucial extension presented here. I therefore start with a short perspective on the Darwinian approach (Section The Darwinian Approach) to avoid possible misunderstandings. Subsequently, the theory is explained in Section Origination Agency and Meaning, and it is connected with biosemiotics in Section Interpretation in Terms of Biosemiotics. In Section On Explanation, Reduction, and the Various Forms of Causation, the issues of reduction and emergence are discussed, and finally, Section Conclusion summarizes the main points of the article.

## The Darwinian Approach

The approach taken here is closely associated with the original Darwinian vision of understanding evolution as a result of the differential reproductive success of organisms that depends on their phenotype.^1^ This vision has often been perceived as implying a materialistic, gene-centred, and deterministic view of life, which excludes genuine agency and meaning.^2^ I will argue below that such an implication is unwarranted, because a slight but far-reaching extension of the basic Darwinian theory can include agency and meaning.

However, it is important to clarify from the beginning how my approach is related to other modern extensions of the evolutionary theory. Modern extensions include interactions between development and evolution, phenotypic plasticity, niche construction, gene-culture coevolution, and a range of sophisticated hereditary mechanisms such as epigenetics and other forms of enhanced evolvability (Laland et al. 2011) and adaptability (Sharov 2014). These factors are considered within the so-called extended evolutionary synthesis (Pigliucci and Müller 2010). Much of this extension is data driven, as more complex evolutionary mechanisms are gradually uncovered. But it is also driven by an implicit concern that the conventional evolutionary view stresses genetic causes too much, to the detriment of other causes that originate from development and behaviour. This apparently motivated Laland et al. (2014) to call it “a struggle for the very soul of the discipline”.

Unfortunately, to the extent that it is an attempt to advocate agency as arising from the organism it appears to be misfiring. Elsewhere (van Hateren 2014a) I argue that causes that seem to originate from the organism do not produce agency if they are merely a result of complex causal loops that are primarily deterministic – with any stochasticity (i.e., randomness) regarded as noise. None of the cogwheels in a clockwork can be a source of agency and meaning, nor can any combination of cogwheels, no matter how complex. The problem with regard to agency is not the apparent origin of causes, but the assumption of determinism. The new, modern mechanisms of evolutionary change can therefore only contribute to agency if they include stochasticity in their causal scheme in a highly specific way (van Hateren 2014a). Below I will focus on the simplest evolutionary mechanism for the emergence of agency, the one that is easiest to understand. However, this does not imply that other processes could not be involved if they similarly entangle deterministic and stochastic forms of causation. I also do not intend to imply that the Darwinian mechanism is the only one producing evolution. But I do claim that the Darwinian mechanism with its extension as explained below is the only one known that is capable of generating agency and meaning. More complex forms of agency and meaning all derive from and depend on this origin.

## Originating Agency and Meaning

Before explaining the process that can generate agency and meaning, I will first make a few general remarks about causation in nature. I use the term ‘causation’ in a very general sense, as referring to any relationship between a cause and its effect (both taken here as changes in time). Thus it includes physical,^3^ mental, and possibly other forms of causation. Note that ‘causation’ is best seen as an idealization (i.e., a mental construct) useful for understanding reality (see also van Hateren 2014b, section 3.3), but it is at the same time taken here to correspond, at least approximately, to actual phenomena in the real world.

**Fig. 1:**
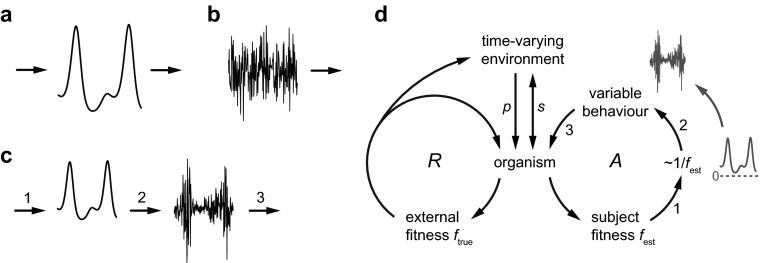
Origin of agency and intrinsic meaning. **a** In deterministic causation, a time-varying variable (representing a system state or property) is caused by (left *arrow*) and causes (right *arrow*) other variables. **b** In stochastic causation, a random variable can start new chains of causation (*arrow*). **c** In modulated stochastic causation, a non-negative deterministic variable (left curve) drives the variance of a stochastic variable (right curve). **d** Behaviour of an organism ultimately depends on Darwinian fitness *f*_true_, which is assumed to be approximated by an internal estimate *f*_est_, made implicitly by the organism itself. It drives the variable component of behaviour that cannot be chosen based on previous learning. The mechanism is evolvable when low *f*_est_ produces large variability and high *f*_est_ small variability (symbolized by ~1/ *f*_est_). The reproductive loop *R* contributes to the population, which is part of an environment that affects the organism's fitness (*arrow p*). The active feedback loop *A* uses modulate stochastic causation (*arrows* 1–3 as in **c**) to gradually produce agency, and intrinsic meaning as embodied in the form of *f*_est_. Agency and meaning depend on feedback through the organism's environment (*arrow s*)

There are two main forms of causation in physical nature. The first, deterministic causation, is illustrated in Fig. 1a. The graph shows the change of a variable, such as a state or some property of a system. This change is caused by other variables (left arrow), and it subsequently causes changes in downstream variables either in the same system or in other systems (right arrow). Causes can be multiple and complex, but the crucial property of a deterministic system is that the change of state remains fully predictable, just like the motions of cogwheels in a clock. In practice, ‘deterministic’ should be interpreted as ‘almost deterministic’ or ‘primarily deterministic’, because real systems always display some noise.

The second main form of causation is stochastic, as illustrated in Fig. 1b. A stochastic process produces changes over time that are not caused by upstream factors but arise spontaneously. This change may then become a starting point of novel downstream causal chains (arrow). Stochastic causation may originate from thermal or quantum noise, from untraceable external disturbances of a system, and from unstable dynamics that amplifies microscopic uncertainties (as in chaos). Stochastic causation implies unpredictability. Stochasticity is ubiquitous in nature in general and in living organisms in particular, from the molecular to the behavioural level (Faisal et al. 2008; Kiviet et al. 2014).

A very specific combination of deterministic and stochastic causation is illustrated in Fig. 1c. It is called ‘modulated stochastic causation’, and is assumed here to play a major role in generating agency and meaning. In this form of causation, one variable (left curve) is caused deterministically by upstream factors (arrow 1). This variable subsequently modulates the variance of a second, stochastic variable (right curve). Finally, the stochastic variable causes changes in downstream factors (arrow 3). For the purpose of presentation, the deterministic variable is shown as changing slowly and the stochastic variable as changing fast, but this is not a necessary property. This type of causation still occurs when the two variables have similar temporal properties, even if it would then be difficult to visualize in a simple graph. Modulated stochastic causation is neither completely predictable (because of the stochasticity), nor completely unpredictable (because the variance of the stochastic variable changes in a deterministic way). Although it can occur in non-living nature, it is in its pure form rather special and therefore likely to be unstable and ephemeral. As will be shown below, it can be stabilized by evolution when it is part of a highly specific and evolvable feedback loop.

Figure 1d explains how modulated stochastic causation can play a crucial role in generating agency and meaning. The left part of the figure represents the basic Darwinian theory of evolution. It consists of a reproductive loop *R*, where organisms reproduce with a rate that corresponds to their fitness (called *f*_true_ below). As a first-order approximation, this rate is defined here as expected number of offspring per unit of time. It is a probabilistic variable that indicates, at each moment in time, how likely it is that an organism will reproduce. Note that it is not identical to the actual number of offspring that an organism gets, which is a stochastic realization of the reproduction rate accumulated over the lifetime of an organism. The fitness indicates, from moment to moment (i.e., instantaneously), how well the organism is doing from the point of view of expected reproduction. It can decrease or increase depending on the circumstances, such as availability of food and mates, or the likelihood or occurrence of a disease. It finally drops to zero when the organism dies. It is important to stress that fitness as used here (both *f*_true_ and its estimate, *f*_est_, as discussed below) are not just simple parameters, but more akin to mathematical functions, which have not only a value but also a form and argument(s). Fitness is in fact a complex dynamical process, with an intricate form (structure) involving many inputs and a single output. The many inputs arise from the environment and internal factors, including the organism's hereditary and behavioural memory. The single output is the expected rate of reproduction. Both the process and its outcome (the rate) are denoted here by *f*_true_ (or by *f*_est_). The intended meaning is usually clear from the context, but in cases of possible misunderstanding I will refer to the value or form of *f*_true_ (or *f*_est_).

Fitness depends on both the organism's properties and the environment, including other organisms. The environment is assumed to vary unpredictably, or at least partly unpredictably, over a wide range of timescales, that is, over evolutionary time as well as within the lifetime of individual organisms. When organisms reproduce, they will produce offspring that is similar, but not identical to themselves. Organisms with fitness exceeding the level required for replacement will increase in numbers exponentially (if the fitness remains high). Their offspring is likely to thrive as well, because environments typically change slowly. But they do change nonetheless, and occasionally quite fast. The variability of offspring properties then implies that some offspring will do better than others. The offspring with higher fitness will reproduce faster, on average. As a result of these differences in reproduction rate, the properties of organisms as distributed over the population will change (i.e., evolve). The environment includes the population and its properties, such as total size and distribution over different types. The *R* loop determines how these properties change over time, which is symbolized in Fig. 1d by the arrow from *R* loop to environment. The population size subsequently affects the reproduction of organisms (as indicated by arrow *p*, which also represents other environmental influences), and thereby their fitness *f*_true_.

The basic Darwinian theory as explained above depends only on deterministic and stochastic causation (or simple combinations), and cannot produce goals and meaning. However, an extension of the basic mechanism is possible that produces such new, emergent phenomena. This extended Darwinian mechanism can be formulated either on the timescale of evolution or on the timescale of the lifetime of individual organisms. The former approach is relevant for changes along lines of descent of organisms (van Hateren 2014a, 2015), whereas the latter is relevant for ontogenetic changes and learning (van Hateren 2014b, 2015). I will focus here on the latter case, where such changes are all summarized by the term ‘behaviour’. Behaviour is interpreted broadly here, including physiological changes within unicellular organisms and phenotypic plasticity.

The right half of Fig. 1d illustrates the main idea of the extended theory. In addition to the true, external fitness *f*_true_ there is also a subject-generated^4^ version of fitness, generated by the organism itself and present in its physiology as an intrinsic process. Like *f*_true_, it is supposed to change over time, and its value is assumed to produce a reasonable approximation of the value of *f*_true_. It is analogous to what one would denote by the term ‘estimate’ in the context of estimation theory. It is therefore called ‘(self-)estimated fitness’, *f*_est_ (van Hateren 2013, 2014a, b, 2015). It should be stressed that the term ‘estimate’ should not be interpreted in terms of human intentions. It is used here to denote the outcome (i.e., the value of *f*_est_) of an internal process (the form of *f*_est_, that is, its physiological realization) that approximates the outcome (the value of *f*_true_) of another process (the form of *f*_true_, that is, the way in which various factors affect *f*_true_). The estimated fitness *f*_est_ does not assume explicit statistical knowledge on the part of the organism, because it can just evolve through regular, undirected evolutionary mechanisms like any other physiological process within organisms (such as colour vision, which does not assume explicit knowledge about electromagnetism). Also, note that *f*_est_ should not be confused with the empirical estimate by an evolutionary biologist based on counting offspring. The estimated *f*_est_ refers here to a real process in an organism, independently of whether scientists exist or not.

The self-estimated fitness affects changes in behaviour in the following way. When *f*_est_ is above the replacement level (the level balancing reproducing and dying), the organism is probably doing well, because *f*_est_ is assumed to reflect *f*_true_. Thus, there is little reason to change behaviour, apart from small changes that may keep options for finding even better behaviour. But when *f*_est_ is below the replacement level, the organism is not doing well and may fail to reproduce or even die. The behaviour should therefore be changed to avoid these outcomes. One possibility is that an organism performs a behaviour that is known (i.e., expected) to work well in given circumstances. Such knowledge may be embodied in genomic memory formed in earlier evolution or in physiological and neural memory formed by previous learning. Such memories were formed when similar circumstances occurred and successful behaviours were found. However, behaviour based on such embodied memories can be performed automatically (i.e., deterministically). It therefore does not directly involve agency, although indirectly it may have been produced by agency originally.^5^ But environmental changes are partly unpredictable, and the consequences of behavioural change are therefore partly unpredictable as well. For this unpredictable part, there is no rule for changing the behaviour. In other words, the change must be random. However, the expected magnitude of the random change (i.e., its variance) should then depend on *f*_est_, because when *f*_est_ is low, then large changes are needed. Large changes in behaviour may initially be disadvantageous especially if the direction of change proves to be wrong. But large changes also increase the probability of finding a behaviour with high *f*_est_. When that happens, the behavioural variability is automatically reduced, because high *f*_est_ implies making subsequent variability small (the *A* loop cycles continually). The subsequent behaviour will then remain in the vicinity of this beneficial behaviour, drifting away only slowly. Simulations show that this way of changing behaviours (small variability when *f*_est_ is large and large variability when *f*_est_ is small) is evolvable (van Hateren 2015). In particular, populations that incorporate this mechanism outcompete populations with a fixed variability of behaviour. The very strong drive provided by fitness is the primary reason why this mechanism is effective: fitness above the replacement level leads to a fast, exponential growth in numbers. It thereby can compensate, on average, for the losses that are produced by large undirected changes and by the time it takes to find behaviours with high *f*_est_.

The inverse relationship between *f*_est_ and behavioural variability is symbolized by ~1/*f*_est_ in Fig. 1d, but the exact form is itself an evolvable property of organisms and may be somewhat different. An assumption of the model is that the value of *f*_est_ has evolved to be sufficiently close to the value of *f*_true_ such that the mechanism would work effectively. That is not a trivial matter (see van Hateren 2015 for a discussion), because *f*_true_ is highly complex. It depends not only on the properties of an organism itself, but also on the unpredictable time-varying environment and on other organisms that are evolving as well. Nevertheless, a perfect match between *f*_est_ and *f*_true_ is not necessary for the mechanism to be advantageous (van Hateren 2015), and the assumption is that even fairly rough versions of *f*_est_ can work well enough.

As an example of how *f*_est_ might approximate the value of *f*_true_, consider a bacterium. Its *f*_true_ depends on a range of external and internal factors, for example the presence of nutrients or toxins, temperature, being prey to amoebae, and internal factors important for maintaining homeostasis. Complex interactions of such factors affect the rate of bacterial reproduction. Some of these factors may be sensed by a bacterium and may be subsequently used, through internal physiological processes, to construct an implicit internal representation and approximation of fitness, *f*_est_. For example, if the bacterium senses a lack of nutrients, this may lower the value of *f*_est_. Similarly, when the bacterium is attacked by amoebae, it may adjust its *f*_est_ by using indicators that are expected to correlate with such an attack. It is important to stress that the value of *f*_est_ is very unlikely to be explicitly represented in the form of a single physiological variable. Rather, it suffices to be present only implicitly, as a distributed and diffuse process operating throughout the physiology of the bacterium. Thus both the form and the value of *f*_est_ are taken to be distributed entities, but nevertheless as real as they come.

Although fitness (both *f*_true_ and *f*_est_) has a single value (a reproduction rate), transforming the single value of *f*_est_ into structural changes of behaviour is far from being simple for real organisms. Each organism has many ways to change its behaviour, but using them all equally (i.e., with equal variance) would not be a good strategy. Instead, behaviours that are associated directly with input factors that strongly determine *f*_est_ should change more than other behaviours. In other words, the partial fitness effects of each input and each behavioural output must be taken into account and be properly weighted. For complex *f*_est_ and complex behavioural possibilities, this will quickly become intractable if one tries to model it explicitly. But it is plausible that a proper association of input and output factors with *f*_est_ can readily evolve through standard evolutionary mechanisms. The implication is that both the process constituting *f*_est_ from its inputs and the process transforming *f*_est_ into a range of behavioural changes are of a distributed nature.

The mechanism of Fig. 1d involves modulated stochastic causation (arrows 1–3 correspond to those in Fig. 1c) operating in a feedback loop (i.e., a loop with cyclical causation,^6^ loop *A* in Fig. 1d). The *A* loop produces goals and agency, as will be argued now. The form of *f*_est_ is defined by which environmental and internal variables an organism uses as predictors of *f*_est_, and in which way it uses them. The form determines which areas of behavioural space (i.e., the possible behavioural repertoire) are associated with low behavioural variability and which areas with high variability. This association is already sufficient, purely for statistical reasons, to drive the behaviour towards the areas with low variability. The word ‘towards’ should not be interpreted too literally here, because the behaviour is not changed into a specific direction – all behavioural changes are random, apart from their variance. But probabilistically, behaviour will diffuse away from areas with high variability more quickly than from areas with low variability, and thus it will tend to stay in areas with low variability. Therefore, it appears to be driven towards such areas. Because low variability is associated with high *f*_est_, high *f*_est_ must then be seen as a genuine goal of an organism. Note that this reasoning does not depend on what exactly *f*_est_ represents. It could represent an arbitrary goal (see Fig. 1b in van Hateren 2015 as an example). But arbitrary goals would not be evolvable through the basic Darwinian mechanism, because they do not specifically promote fitness and probably even reduce fitness, as striving for goals generally carries costs. In other words, the only goal that is evolvable and stable in the long run is fitness itself, *f*_true_. Consequently, *f*_est_ must be an approximation of *f*_true_, because otherwise the mechanism would not have evolved. There is no guarantee that *f*_est_ will remain an approximation of *f*_true_ when circumstances change, but a mismatch would lead to a disadvantage relative to other organisms with a better form of *f*_est_. Thus a persistent mismatch would presumably lead to extinction, and would have done so in the past. It is therefore likely that *f*_est_ has evolved to become fairly robust against common disturbances.

Although striving for high *f*_est_ is the overall goal of an organism, in practice this goal will consist of a large number of sub-goals. Such sub-goals can be seen as resulting from a partitioning of the form of *f*_est_, that is, a partitioning of the process constituting *f*_est_ into sub-processes. Together, these sub-processes, and the sub-goals they represent, serve the general goal of high *f*_est_. Partitioning of *f*_est_ into effective and coherent sub-processes is likely to facilitate improving the form of *f*_est_, through evolution or learning, and is therefore likely to be evolvable.

Apart from establishing *f*_est_ as a genuine goal, the *A* loop also produces agency, because the causation that results from the feedback loop is rather special. The modulated stochastic causation already intermingles deterministic and stochastic factors (*f*_est_ and the behavioural variability, respectively), but the loop strongly amplifies this effect. Each time the loop is traversed (which happens continually), *f*_est_ and the stochasticity become further entangled. First, the value of *f*_est_ determines the behavioural variability and the stochastic outcome determines a new behaviour; then, the new behaviour leads to a new value of *f*_true_ and therefore a new value of *f*_est_. In the next pass through the *A* loop, the new value of *f*_est_ again determines behavioural variability, and so on and so forth. Eventually, there is no way to separate causation into deterministic and stochastic components. The details of the behavioural trajectory are unpredictable because of the stochasticity, but the overall direction of the trajectory depends on the goal, namely high *f*_est_. The behaviour therefore combines a certain spontaneity (in the form of stochasticity) with a certain deliberateness (in the form of striving for high *f*_est_). Such a combination is the signature of agency, at least an elementary form of agency (elsewhere I call it ‘active causation’, van Hateren 2015). The behavioural trajectory is driven by an internal goal (high *f*_est_), but the trajectory is not fully determined, for two reasons. First, because of the stochasticity in the *A* loop, as discussed above. Second, the form of *f*_est_ is not fixed, neither in evolution nor within the lifetime of an individual organism. This is because there are many different forms of *f*_est_ that are approximately equivalent in terms of how closely they can approximate the value of *f*_true_. Such different forms and their improvements are evolvable and learnable as well. They may be accessible through hereditary and behavioural variability, but also more deliberately through a dialogue between or within organisms (van Hateren 2014b).

By internalizing the external *f*_true_ as *f*_est_ via the *A* loop, the organism obtains a genuine goal and genuine agency. Having a goal and agency implies that the goal is important to the organism and thereby assigns value to the goal. In other words, the behaviour becomes meaningful. Because this meaning is generated within the organism, I have elsewhere coined the term ‘intrinsic meaning’ for it (van Hateren 2015). The emergence of meaning suggests that the current theory can be interpreted in terms of semiotics (see Interpretation in Terms of Biosemiotics).

## Interpretation in Terms of Biosemiotics

### The Semiotic Triad

Biosemiotics involves the study of meaning in biological systems, and amongst its intellectual roots is semiotics. One of the most popular systems for describing signs and their meaning is the triadic one promoted by Peirce (2010). This system is often used for analysing meaning in a linguistic context (e.g., Chandler 2007), but it can also be applied to meaning in biology (e.g., Hoffmeyer 2012). My purpose here is to show that the meaning-generating theory described above can be represented as a triad. Although this suggests a resemblance to the Peircian triad, it is left to future analysis to establish how deep that resemblance goes.

**Fig. 2:**
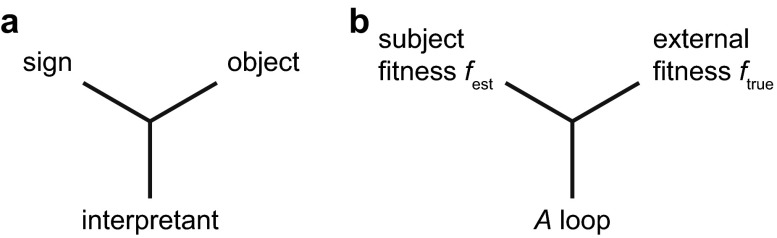
Semiosis. **a** Standard Peircian triad, where a sign (sign vehicle) is connected to its (semiotic) object through an interpretant, which interprets the sign and produces the meaning of the triad. **b** Tentative interpretation of the theory of Fig. 1d as a (primordial) semiotic triad, where the *A* loop interprets *f*_est_ as an estimate of *f*_true_, resulting in enhanced fitness and the emergence of agency and meaning

Figure 2a shows how the basic Peircian triad can represent signification, the overall process of producing meaning. It consists of three elements that become mutually related, as symbolized by the lines joined in the centre. The sign (or sign vehicle) is called representamen by Peirce, because it represents. It is connected to an object (the semiotic object to which the sign vehicle refers) by the interpretant. The interpretant produces the interpretation of the sign and thereby, more generally, the meaning of the overall process. A typical example of a sign is smoke that is connected to its object, fire, through an interpretant that consists of the idea that smoke usually indicates fire. Smoke is then a sign of fire.

Figure 2b shows how the mechanism of Fig. 1d can be tentatively interpreted as a (primordial) semiotic triad. The subject-generated *f*_est_ refers to the external *f*_true_ through the meaning-generating *A* loop. The *A* loop implicitly interprets *f*_est_, and by doing so enhances the organism's fitness. This loop is the primary source of meaning, and because of the dynamical and stochastic nature of the mechanism, it generates agency as well. The organism as in Fig. 1d then gets the role of semiotic agent, which in effect uses the semiotic triad. The three entities that occupy the corners of Fig. 2b are far from simple. The *A* loop is an unusual stochastic feedback process, and *f*_est_ and *f*_true_ are complex processes (with reproductive rates as outcome) that keep changing because of the feedback and because of changes in the environment and organism.

As argued elsewhere (van Hateren 2014b), the relationship between *f*_est_ and *f*_true_ can also be seen as a primordial form of intentionality (‘aboutness’, the property of standing for or referring to something else; *f*_est_ is about *f*_true_). In a sense, the form of *f*_est_ represents all the organism knows about its situation (as objectively represented by the form of *f*_true_), which is similar to the concept of knowledge as discussed in Kull (2009). Both *f*_est_ and *f*_true_ are complex processes with many inputs and at least some of their components are likely to be related. This is likely, because only with related components *f*_est_ can approximate the value of *f*_true_ across a wide range of circumstances. An example of a related component is glucose surrounding a bacterium. Its presence may partially determine *f*_true_, and the sensing of glucose by the bacterium may partially determine *f*_est_. Such related components are part of another semiotic triad by themselves, with as interpretant the fractional role glucose plays in the *A* loop. This more detailed level of semiosis may in fact be more readily amenable to Peircian analysis than the rather abstract general level of *f*_est_ and *f*_true_.

One difference between the triad of Fig. 2b and semiotic triads as used for linguistic analysis is that the current triad is not in the middle of a web of interrelated meanings. Rather, it is a source of such a web (in addition to being interconnected with the triads of other organisms). Neither *f*_true_ nor the *A* loop can be expanded into further triads. But *f*_est_ can be expanded because it consists of a myriad of sub-goals that together contribute to the overall goal of high *f*_est_. Such sub-goals form the bulk of specific meanings as studied in biosemiotics, for example when assigning meaning to certain molecular processes serving an organism (Barbieri 2008). The reason for differentiating the triad of Fig. 2b from linguistic triads is that it presumably lies at the root of all meaning and agency (van Hateren 2014b, 2015). There are no meanings below this level, and thus, this triad is different from those of the high-level signs of language.

### Concordances and Discordances with Eight Theses of Biosemiotics

In Kull et al. (2011), the conceptual basis and basic principles of the field of biosemiotics are summarized in the form of eight theses. It is therefore interesting to see to what extent the approach presented here is consistent with these principles, as discussed below (theses I-VIII are all cited from Kull et al. 2011).

“I. The semiosic/non-semiosic distinction is co-extensive with life/non-life distinction, i.e., with the domain of general biology”. This is consistent with the argument in van Hateren (2013), where the *A* loop (steering either hereditary or behavioural change) is viewed as producing the agency and meaning of life.

“II. Biology is incomplete as a science in the absence of explicit semiotic grounding”. In van Hateren (2014a) I argue that the hereditary version of my theory produces an inherent teleology within organisms, with *f*_est_ and the *A* loop generating goals and, at the behavioural level, agency (van Hateren 2014b).

“III. The predictive power of biology is embedded in the functional aspect and cannot be based on chemistry alone”. When all organisms have agency and intrinsic meaning, prediction must utilize their implicit goal-directedness as one of the three primary factors (along with environment and heredity/physiology). Sometimes the latter two factors (e.g., a harsh winter or genetic disease) may determine biological outcomes without also being caused by the organism's agency and goals. But usually, biological outcomes also depend on (are partly caused by) agency and goals, for example, when an animal deliberately migrates to a new territory. Although the value of *f*_est_ is ultimately produced by a physiological process, that process can only be interpreted if it is recognized as a key component of the stochastic mechanism from which agency, goals, and meanings emerge. Thus the intention to migrate is a real phenomenon that must be included in a complete explanation of why the animal migrates, including predictions of such behaviour.

“IV. Differences in methodology distinguish a semiotic biology from the non-semiotic one”. The current approach does not specifically address methodology, but it is at least compatible with this thesis. Meaning is often implicitly used for analysing living systems in terms of using and processing information. Examples are cases where genetic information is interpreted (for a review of biosemiotic interpretation see El-Hani et al. 2006) and where sensory and neural processing is viewed as a form of information processing. The specificity of the current theory may help to distinguish information that is functioning as meaningful to the organism itself from information that is merely used as an analysis tool by the investigator (and therefore may be only meaningful to the investigator rather than to the organism).

“V. Function is intrinsically related to organization, signification, and the concept of an autonomous agent or self”. This thesis is closely related to the thesis of autopoietic theory (e.g., Thompson 2007) that autonomy and self-maintenance as such represent meaning. I am critical of this viewpoint (see also extended discussions in van Hateren 2013, 2014b), because self-maintenance may be purely deterministic (or have stochasticity without an *A* loop) and thus may produce no agency. Self-organization is sometimes seen as the source of autonomy, but self-organization is quite common in nature, occurring whenever systems have unstable and self-reinforcing dynamics (e.g., spontaneously generated tornadoes). Furthermore, maintaining the self as an autonomous unit can only be regarded as normative (implying goals and meaning) when the additional (tacit) assumption is made that existing is better than not existing (van Hateren 2014b). Such an assumption is unwarranted, unless there is already an *A* loop. I also do not agree with the thesis “Evolution presupposes function, rather than vice versa” (Kull et al. 2011, p. 32) if the term ‘function’ is regarded as normative. The basic Darwinian theory of evolution by natural selection could, in principle, work without the extension with an *A* loop. It would lead to self-reproducing systems without agency and meaning, and could not produce systems with consciousness. However, this is a hypothetical case, because the extension provides an evolutionary advantage and presumably evolved very early on (see van Hateren 2013 for a discussion). Moreover, it is conceivable (but nearly impossible to prove) that without an enhanced fitness-driven selection (*f*_est_ amplifies *f*_true_) the overall drive would be too weak, in practice, to let proto-life get off the ground or to prevent it from becoming extinct at an early stage.

“VI. The grounding of general semiotics has to use biosemiotic tools”. This thesis is consistent with the approach in van Hateren (2014b), which views more complex forms of meaning, such as associated with human consciousness and language, as emerging from more basic forms of meaning that are also present in non-human species. The word ‘grounding’ acknowledges the possibility of emergence and the subsequent necessity to use novel concepts (e.g., in the social sciences and humanities). This would be similar to the present approach, which uses tools from the natural sciences to ground biosemiotics, leading to novel concepts like agency and meaning.

“VII. Semiosis is a central concept for biology – however, it requires a more exact definition”. The *A* loop and its elaborations can be seen as a defining, prototypical model, as a valid proxy for a verbal definition. It incorporates several of the seven specific criteria mentioned by Kull et al. (2011, pp. 36–38), in particular agency, normativity, teleo-functionality (e.g., van Hateren 2014a), form generation (stochastic, meaningful changes can explore novel forms as retained in hereditary and behavioural memory), and inheritance of relations (e.g., van Hateren 2014b). Categorization is not specifically included, but is consistent with the theory as a property that can evolve, e.g., as a way to stabilize long-term memory. It can thereby support meaning, including its extension to high-level symbolic systems (van Hateren 2014b). I believe there is discordance with the final criterion, namely that a sign vehicle must be insulated from the dynamics that it constrains. This is similar to the notion that the controlling system must be separated from the controlled system (Pattee 2008). However, this requirement of a strict separation of initial conditions (doing the controlling) and laws (subsequently determining the fate of the controlled system) implicitly assumes systems described in a deterministic manner. When the actual physical system is not deterministic, but partly stochastic in the specific way of the *A* loop, it is no problem to have controller and controlled being part of the same dynamics. A key point here is that agency and meaning are not instantaneous, but only gradually build up statistical significance (see van Hateren 2014b, 2015). The entanglement this implies makes it impossible to separate controller and controlled.

“VIII. Organisms create their umwelten”. The Umwelt is a concept that comes from von Uexküll (1982), who suggested that organisms perceive and interpret the world into which they are embedded by generating internal meanings. The concept of Umwelt is closely associated with the form of *f*_est_, the means through which an organism attaches meaning to everything it implicitly takes to be relevant for its *f*_true_. The organism actively interacts with its world, modifying it and being modified by it. The double-headed arrow *s* in Fig. 1d, which indicates a feedback loop through the environment, is similar to von Uexküll's functional cycle. The result is that the organism lives in a semiotic niche (Hoffmeyer 2008b) that depends on the organism's own interpretations and coexists with the ecological niche. However, the semiotic niche is still strongly connected to the ecological niche, because *f*_est_ is tied to *f*_true_. Therefore, the word ‘create’ in thesis VIII should not be interpreted as ‘freely construct’, that is, the construction of an Umwelt is neither completely free nor completely determined.

The conclusion from the above discussion is that there is clearly a considerable overlap between the theory presented in this article and standard biosemiotic notions. Apart from a minor discordance with part of thesis VII, there is a stronger discordance with thesis V with respect to the origin of agency and meaning. The current theory partially agrees with thesis V to the extent that it also requires that organisms have enough autonomy such that fitness (i.e., *f*_true_) takes a form that enables evolution. But such autonomy is only necessary, not sufficient for normative functions. Normativity and intrinsic goal-directedness are proposed here to emerge from *f*_est_ and the stochastic mechanism of the *A* loop, which produces agency as well. Agency as understood here is in fact largely consistent with its typical use in biosemiotics (Tønnesen 2015), where the “core attributes of an agent include goal-directedness, self-governed activity, processing of semiosis and choice of action” (see also van Hateren 2014b). For most species, the expression “choice of action” is probably a bit too strong, because choosing seems to presuppose sharp categorization. I rather prefer to call it “some behavioural freedom” (van Hateren 2015), where behaviour is interpreted broadly to include also processes within plants and unicellular organisms. But apart from wording, it points to a similar concept.

## On Explanation, Reduction, and the Various Forms of Causation

Some readers may worry that the theory as presented in Fig. 1d is inherently reductionistic, i.e., that it tries to reduce meaning and agency to purely physical processes. I will argue that such an interpretation is wrong, and that this theory in fact explains the phenomenon of emergence – that is, the emergence of meaning and agency. Although emergent phenomena arise from simpler ones, they subsequently displace the simpler phenomena as components that produce and explain higher levels. For explanations, such displacement is not facultative, but inescapable, as will be argued below.

Explanation often involves showing how simple phenomena give rise to more complex ones. When this works perfectly, one might say that a more complex phenomenon is reduced to and fully defined by the simpler ones, including their configuration. For example, the fact that a chair does not break down when one sits on it is fully explained by the strengths of the seat and legs, and by how they are connected. It is highly plausible that complex phenomena are always produced by dynamical configurations of simpler phenomena. A simple argument for this is the following. It is quite certain by now that not long after the beginning of the universe the temperature was so high that there were only elementary particles, not even atoms yet. That means that all more complex phenomena we can observe today – atoms, stars, planets, life, meaning, and consciousness – must have coalesced, through time, from simpler precursors. Reduction can be regarded as an attempt to reconstruct these simpler precursors and their subsequent coalescence, preferably not by simulating the entire process through time but by using symbolic simplifications and short-cuts.

However, there are two major problems for such reconstructions. Both come from the way human thinking works: explanation for humans requires symbolic representation (Deacon 1997). Systems of symbolic representation, in particular mathematical systems, can be highly effective for describing fairly simple or restricted parts of nature. But they quickly become intractable when systems become too complex. This is the first major problem for reduction. For example, Laughlin and Pines (2000) argue that fully explaining proteins in terms of quantum physics is infeasible in practice, and will most likely remain so in the future. The new, empirically observed properties of such molecules must therefore be taken as a starting point for a new discipline, organic chemistry. Organic chemistry is then not reducible to quantum physics, and it is, as a science, not less basic (Anderson 1972; Laughlin and Pines 2000).

The second major problem for reduction is even more fundamental. Mathematical systems can only accurately represent the dynamics of deterministic systems. Stochastic phenomena can only be described in a roundabout, probabilistic sense, by using indirect constructs such as probability distributions (van Kampen 2007). It therefore may seem rather problematic that reality appears to be, deep down, strongly stochastic, as shown by modern physics. Fortunately, the stochasticity in nature often averages out and fades away to such an extent that mathematical description and prediction are quite feasible (e.g., for the motion of the planets in the solar system) or at least somewhat feasible (e.g., for the weather).

In contrast, the *A* loop of Fig. 1d has properties that seem to make mathematical description and prediction as infeasible as possible.^7^ Stochasticity gets a crucial role, in a way that makes it impossible to let it average out or fade away. Stochasticity as driven by *f*_est_ is a prime factor in the causation, with lasting effects on future stochasticity. The *f*_est_ and stochasticity at a particular point in time depend on the entire history of *f*_est_ and all previous stochastic outcomes, because the *A* loop entangles these factors and the result is stored in effectively permanent structural changes (memory). Hereditary and physiological properties are retained over evolutionary time in the form of DNA and the cellular system of organelles, which are indeed partly traceable to initial storage billions of years ago. Similarly, development and learning establish memory that is often permanent within the lifetime of individual organisms. Unfading memory means that the *A* loop follows ever-changing, newly created trajectories through newly created parts of hereditary and behavioural space. Consequently, it produces non-ergodic dynamics, with future spaces undefinable in the present.

A further factor making complete mathematical description impossible is the complexity of the feedback loop and the factors involved. In particular, *f*_est_ is presumably produced by physiology that – directly or indirectly – involves much, if not all of the organism, because the functioning of the entire organism is the prime factor for its fitness. Worse yet, *f*_est_ cannot be understood in isolation, but only as an approximation of *f*_true_ and by how that affects the *A* loop. A model of the *A* loop therefore also requires a model of *f*_true_. Although *f*_true_ is an objective variable and process (implicitly produced by nature happening), a scientist trying to model it would need detailed knowledge of how a complex organism functions within a complex environment that includes a complex biosphere. Exact knowledge of *f*_true_ is clearly not possible in realistic situations. Moreover, *f*_true_ is not independent of the *f*_est_ (and its implied agency) of the organisms present in the environment, which leads to complex causal loops at an ecological level.

From the above discussion we have to conclude that the functioning of the *A* loop cannot be reduced, not even in principle, to its components and how they are configured. Similarly, its properties cannot be derived in any detail. Nature does produce them, but there is no way to model that process completely. Highly simplified versions of the loop (such as used for the simulations in van Hateren 2015) can provide some insight into what is happening, but realistic versions will remain beyond reach. It is plausible that the loop produces the new phenomena of agency and meaning, because of the loop's structure and the way by which it lets *f*_est_ drive stochasticity. However, these phenomena have to be seen as novel, as emerging *de novo*. As usual in science (Anderson 1972), such phenomena can subsequently be used as the starting point for a higher-level discipline or disciplines. Nevertheless, for living organisms the physical and meaningful will need to be integrated. Whereas the left half of Fig. 1d is mostly physical, it is influenced by the *f*_est_ of the organisms in the environment. The right half of Fig. 1d is the origin of agency and meaning, but it is at the same time physical because it depends on the organism's physiology.

The current analysis started from physical causation in the form of deterministic and stochastic causation. The combined form of modulated stochastic causation still belongs to the physical world. However, the causation produced by the *A* loop, called active causation, already belongs exclusively to life. It is an elementary form of agency, closely related to what is elsewhere called ‘semiotic causation’ (Hulswit 2002; Hoffmeyer 2008a), i.e., the bringing about of effects through interpretation. The new form of causation has emerged from the highly specific combination of purely physical causation as occurring in the *A* loop. But having emerged, it can no longer be described purely in physical terms. It depends on goal-directedness, meaning, and agency, which are phenomena that are not present in the physical world of non-living matter. As a result, changes in the world of life can only be understood from three rather than two basic forms of causation: deterministic, stochastic, and active/semiotic. The latter form can subsequently evolve into increasingly complex forms of agency (van Hateren 2014b), including variants that might be described by a term like mental causation (or intentional causation, as in Searle 2013).

## Conclusion

Meaning and agency can be generated by an extension of the basic Darwinian theory of evolution. This extension consists of a feedback loop in which a self-estimated version of the organism's fitness, *f*_est_, modulates stochasticity as driving behavioural change, with *f*_est_ under selection pressure to conform to the organism's true fitness, *f*_true_. The theory resembles the Peircian semiotic triad, with *f*_est_ in the role of sign, *f*_true_ as its object, and the feedback loop as interpretant. Moreover, the theory is mostly consistent with standard notions as developed in biosemiotics. The theory results in two novel properties, agency and goal-directedness (implying meaning) that emerge from the dynamics of the proposed mechanism. These properties are novel primitives, irreducible to a purely physical description.

## Footnotes

1 A phenotype is the actual form (its total of characteristics) through which an organism interacts with the world.

2 Throughout this article, the term ‘meaning’ is used in a general sense as in ‘the meaning of an action’, rather than in the more specific sense as in ‘the meaning of a word’.

3 The term ‘physical’ as used in this article refers to anything conventionally studied by the natural sciences, in particular non-living matter, but also the material aspects of life. It excludes mind, primarily mental processes, agency, value, and meaning.

4 I deliberately use ‘subject-generated’ rather than ‘subjective’ here, because the meaning of the latter is often associated with consciousness and idiosyncrasy, whereas the subject-generated fitness is constrained by the requirement that it conforms reasonably well to *f*_true_, which is an objective variable.

5 Original agency does not imply current agency. As a crude analogue, appliances such as autopilots and robotic lawn mowers exist and work because of the agency and goals of their builders and users, and in a sense they empower their users’ agency and goals. But they are not a source of agency and goals themselves.

6 The term ‘feedback’ is used here in the general sense of any cyclic or mutual causation, thus not limited to stabilizing (negative) feedback.

7 In addition, the theory (in particular the mechanism of the *A* loop in Fig. 1d) is of a form that makes intuitive comprehension difficult, because it combines two concepts – stochasticity and feedback – that imply highly counter-intuitive forms of causation. Whereas stochasticity has the perplexing property of producing uncaused change, feedback has the perplexing property of confounding cause and effect (when A causes B and B causes A, which is then cause and which effect?). Moreover, the mechanism is driven by Darwin's theory (as represented by the *R* loop in Fig. 1d), which is in itself hard to comprehend intuitively.
